# RNA expression profiling of peritoneal metastasis from pancreatic cancer treated with Pressurized Intraperitoneal Aerosol Chemotherapy (PIPAC)

**DOI:** 10.1515/pp-2024-0001

**Published:** 2024-06-03

**Authors:** Sönke Detlefsen, Mark Burton, Alan P. Ainsworth, Claus Fristrup, Martin Graversen, Per Pfeiffer, Line S. Tarpgaard, Michael B. Mortensen

**Affiliations:** Department of Pathology, Odense University Hospital, Odense, Denmark; Odense Pancreas Center (OPAC) and Odense PIPAC Center (OPC), Odense University Hospital, Odense, Denmark; Department of Clinical Research, Faculty of Health Sciences, University of Southern Denmark, Odense, Denmark; Department of Clinical Genetics, Odense University Hospital, Odense, Denmark; Clinical Genome Center, University of Southern Denmark, Odense, Denmark; Department of Surgery, Upper GI and HPB Section, Odense University Hospital, Odense, Denmark; OPEN–Open Patient Data Explorative Network, Odense University Hospital, Region of Southern Denmark, Denmark; Department of Oncology, Odense University Hospital, Odense, Denmark

**Keywords:** pancreatic cancer, Pressurized Intraperitoneal Aerosol Chemotherapy (PIPAC), peritoneal metastasis, Peritoneal Regression Grading Score (PRGS), RNA profiling, transcriptomics

## Abstract

**Objectives:**

Pressurized Intraperitoneal Aerosol Chemotherapy (PIPAC) is an experimental treatment option in peritoneal metastasis from pancreatic cancer (PM-PC). Aims were to examine mRNA profile of fibrosis due to response after systemic chemotherapy and PIPAC (Regression) compared to treatment-naïve PM-PC and chronic cholecystitis–related peritoneal fibrosis (Controls).

**Methods:**

Peritoneal biopsies (PBs) from PM-PC patients who had undergone systemic chemotherapy and PIPAC were evaluated with Peritoneal Regression Grading Score (PRGS). We extracted RNA from PBs with Regression (PRGS 1, n=11), treatment-naïve PM-PC (n=10), and Controls (n=10). Profiling of 800 mRNAs was performed (NanoString nCounter, PanCancer Immuno-Oncology 360 (IO-360) and 30 additional stroma-related mRNAs).

**Results:**

Regression vs. PM-PC identified six up-regulated and 197 down-regulated mRNAs (FDR≤0.05), linked to TNF-α signaling via NF-kB, G2M checkpoint, epithelial-mesenchymal transition, estrogen response, and coagulation. Regression vs. Controls identified 43 significantly up-regulated mRNAs, linked to interferon-α response, and down-regulation of 99 mRNAs, linked to TNF-α signaling via NF-kB, inflammatory response, epithelial-mesenchymal transition, KRAS signaling, and hypoxia (FDR≤0.05).

**Conclusions:**

In regressive fibrosis of PM-PC after systemic chemotherapy and PIPAC (Regression), downregulation of mRNAs related to key tumor biological pathways was identified. Regression also showed transcriptional differences from unspecific, benign fibrosis (Controls). Future studies should explore whether mRNA profiling of PBs with PM from PC or other primaries holds prognostic or predictive value.

## Introduction

The prognosis of patients with peritoneal metastasis (PM) from pancreatic cancer (PC) is poor, with a median survival after systemic combination chemotherapy in highly selected patients of 7–8 months [1]. Pressurized Intraperitoneal Aerosol Chemotherapy (PIPAC), with or without systemic chemotherapy, is a treatment option in PM-PC patients [2], [3], [4], [5], [6]. PIPAC is an experimental treatment, and randomized controlled phase three trials are lacking, yet some are ongoing [7], [8], [9], [10], [11]. PIPAC is administered every 4 to 6 weeks, and peritoneal quadrant biopsies (PBs) taken prior to each treatment are used for histological response evaluation [2, 12], [13], [14], [15]. Regressive fibrosis is the main histological feature of therapy response [4, 5, 13, 14, 16]. The transcriptomic profile of therapy response in PM-PC has not been investigated. PM is rarely re-biopsied after treatment, but patients receiving PIPAC treatment represent a unique opportunity to study therapy-induced changes in RNA expression.

The general response evaluation method in PC patients, computed tomography-based Response Evaluation Criteria in Solid Tumors (RECIST), is of limited value in PM [17] but still used in several published and ongoing studies [10, 11]. The histological Peritoneal Regression Grading Score (PRGS) seems at present to be the most promising tool for evaluating treatment response in PIPAC-treated PM [17]. Other variables for evaluation of treatment response in PM are survival, peritoneal lavage cytology, peritoneal cancer index (PCI), eligibility for radical surgery after PIPAC, and quality of life (QoL) [5, 15, 17], [18], [19]. The value of magnetic resonance imaging (MRI) or fluorodeoxyglucose positron emission tomography combined with CT (FDG PET-CT) in this setting is currently unknown [17].

The PRGS is a four-tiered scoring system and based on the relative amounts of residual tumor and regressive histological features [13, 14, 16]. The most important histological feature of regression in PM-PC is fibrosis, often accompanied by varied numbers of inflammatory cells and foamy macrophages. Regressive fibrosis is characterized by reduced numbers or absence of cancer cells. A few recent studies demonstrated that PRGS holds prognostic value, either alone or in combination with peritoneal lavage cytology [2, 12, 15]. When using clips-marking of biopsy sites, the same areas can be re-biopsied after treatment [20]. This makes PBs an interesting source for the study of treatment-related transcriptional changes in PM. Only one previous study examined gene expression patterns after application of chemotherapy in patients with PM, using a 22-gene panel in patients with mainly PM from ovarian cancer [21]. Studies looking at a larger panel of mRNAs, such as RNA sequencing or RNA expression profiling of hundreds of different mRNAs known to play a role in cancer biology, are currently lacking.

Our primary aim was to examine dysregulated mRNAs in fibrosis due to therapy response (Regression), compared to treatment-naïve PM-PC. Secondary aim was to identify genes that are differently expressed in the Regression group compared to benign peritoneal fibrosis (Controls).

## Materials and methods

### Clinical data and inclusion of specimens

**Figure 1:**
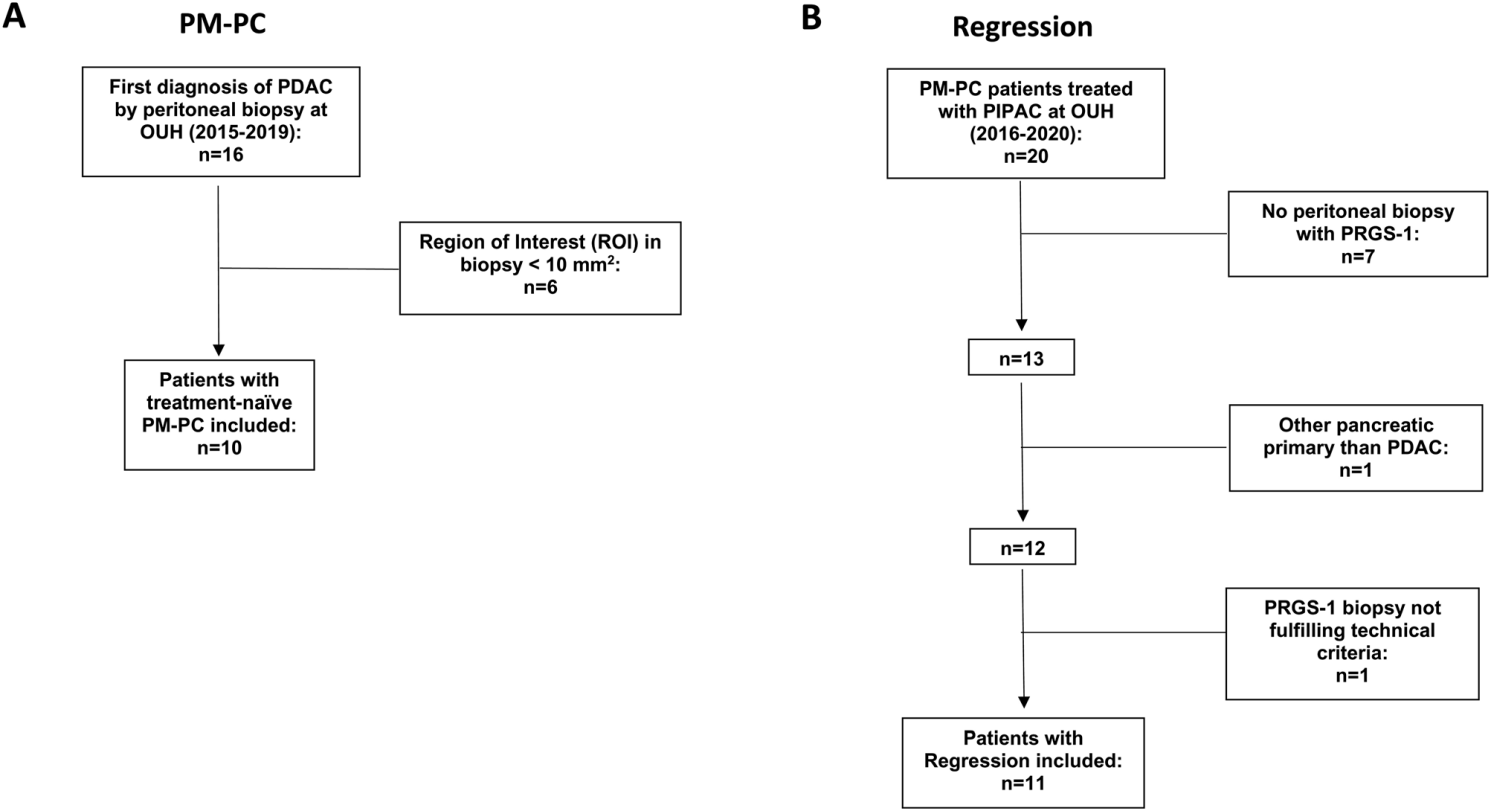
Flow chart diagrams of patient inclusion. (A) Inclusion of patients with chemotherapy-naïve peritoneal metastasis from pancreatic cancer (PM-PC). (B) Inclusion of patients with PM-PC and peritoneal biopsies showing complete regression after systemic chemotherapy and PIPAC (Regression). OUH, Odense University Hospital; PDAC, pancreatic ductal adenocarcinoma; PRGS, Peritoneal Regression Grading Score.

We included peritoneal biopsies (PBs) from patients with treatment-naïve PM-PC, using the following inclusion criteria: The diagnosis (PM from pancreatic ductal adenocarcinoma) was established on a PB, typically a frozen section as part of a laparoscopic evaluation, performed at Odense University Hospital (OUH), Denmark, in the period from 01.03.2015 to 28.02.2019. The patient had to be treatment-naïve. Region of interest (ROI) had a size of ≥20 mm^2^. This resulted in the inclusion of 10 patients with PM-PC (Figure 1A).

We included PBs from patients with regression of PM-PC after systemic chemotherapy and PIPAC treatment (Regression group), who had received their first PIPAC treatment in the period 01.01.2016–30.06.2020, using the following criteria: One PB with complete regression (PRGS-1) was included, the patient had at least one PIPAC treatment prior to the included PB, and the ROI had a size of ≥7 mm^2^. First, PBs taken prior to PIPAC-3 were evaluated, followed by those obtained prior to PIPAC-2, PIPAC-4, and, lastly, PIPAC-5. The first PB fulfilling these criteria was included in the Regression group. Of note, the entire quadrant PB set did not have to show complete regression (PRGS-1). This resulted in the inclusion of 11 patients with Regression (Figure 1B).

To be able to compare possible transcriptomic differences between Regression and chronic peritonitis–related fibrosis (Controls), we included 10 cholecystectomy specimens with chronic cholecystitis and severe subperitoneal fibrosis. These were included consecutively, starting with patients who had undergone cholecystectomy at OUH from 01.01.2016 onward. The ROI had a size ≥20 mm^2^.

This study complies with the World Medical Association Declaration of Helsinki regarding ethical conduct of research involving human subjects. It was approved by the Scientific Ethics Committee of the Region of Southern Denmark (project-ID S-20200122) and by the Data Protection Agency of Region of Southern Denmark (journal-no 20/51469). We ensured that patients had not advocated against the use of their tissue in the Danish registry for the use of tissue in research (“Vævsanvendelsesregisteret”).

### The Peritoneal Regression Grading Score (PRGS)

PBs were assessed by the four-tiered PRGS to evaluate the histological response to therapy in patients with PM [14]. Scoring was in all cases performed by the same pathologist (SD) who had an interest in peritoneal pathology. PRGS is based on the presence of residual tumor cells in relation to the extent of regressive features. Major histological features of regression are fibrosis, inflammation, hyalinosis, acellular mucin pools, ischemic necrosis, accumulation of macrophages, multinucleated giant cells, and granulomas. PRGS-1 corresponds to a complete regression with absence of tumor cells; PRGS-2 to a major histological response; PRGS-3 to a minor histological response; and PRGS-4 to metastatic tumor only, with a lack of histological response to therapy [13]. PRGS is given for each PB separately, in addition to a mean score for all biopsies belonging to a given quadrant PB set.

### RNA extraction

We mounted 10 µm-thick FFPE sections from the PM-PC, Regression, and Control cases on Superfrost PlusTM Adhesion Microscope Slides (ThermoFisher Scientific, Braunschweig, Germany, J1800AMNZ). We aimed to include a total area of 100 mm^2^, and the number of sections was adjusted accordingly. Macrodissection was done when appropriate. Sections were dried overnight prior to deparaffinization. Then, RNA extraction was performed using High Pure FFPE RNA isolation kit (Roche diagnostics GmbH, Mannheim, Germany, 06650775001) according to the Prosigna protocol. RNA concentration and A260/280 were determined using NanoDrop One (Thermo Fischer Scientific, Wilmington, DE, USA). All samples were immediately stored at −80 °C.

### mRNA expression profiling and data processing

For the 31 specimens, mRNA gene expression levels were assessed using the PanCancer immune-profiling panel, Immuno-Oncology 360 (IO-360, NanoString Technologies, Seattle, WA), designed to give a unique 360° view of gene expression in the tumors. The panel consists of 750 genes (and in addition 20 housekeeping genes), falling into eight functional categories: (1) tumor immunogenicity, (2) tumor sensitivity to immune attack, (3) inhibitory immune mechanisms, (4) stromal factors, (5) inhibitory metabolism, (6) antitumor immune activity, (7) inhibitory immune signaling, and (8) immune cell population abundance. A total of 30 custom genes were added, related to fibroblasts, endothelial cells, and extracellular matrix (ECM), as described previously [22]: *ACTA2*, *ANO1*, *CALD1*, *CD34*, *CEACAM5*, *COL3A1*, *COL4A1*, *CYGB*, *FN1*, *GPC1*, *HAS2*, *INS*, *KCNH2*, *KRT7*, *KRT8*, *LGALS1*, *MME*, *MUC1*, *NES*, *PDPN*, *POSTN*, *PRSS1*, *S100A4*, *SLC16A3*, *SMAD4*, *SPARC*, *SYP*, *TNC*, *VCL*, and *VIM* [23].

RNA samples were aligned with a synthetic panel standard and hybridized in a 12-strip tube well with gene-specific probes (reporter and capture) for 16–21 h at 65 °C, following the manufacturer’s protocol. Subsequently, the target-probe complexes were read and counted by scanning of 550 fields of view (FOV) using the nCounter Digital Analyzer (NanoString Technologies, Seattle, WA) [24]. Raw digital counts of expression were exported to the nSolver v4.0 software (NanoString) for downstream analysis, following the manufacturer’s protocol.

In nSolver v. 4.0, the data were investigated to check quality of reads according to manufacturer’s instructions. Genes with an expression level below the average count of negative controls plus two standard deviations were considered undetected. After normalization against chemical positive controls, data were exported from nSOLVER 4.0 software. The subsequent calculations and analyses were performed using the open source R-environment (v. 4.3.2).

### Statistics

Values were given as medians with range, means with standard deviation (SD), or percentages where appropriate. Comparisons between gene counts for Regression vs. PM-PC and Regression vs. Controls were performed using the open source R-environment (R version 4.3.2) (http://cran.r-project.org/). Gene expression data were converted to counts-per-million (CPM), normalized using the trimmed mean of M-values (TMM) method and converted to log2 scale, and based on all genes, the samples were analyzed by unsupervised hieararchial clustering, and expression levels were visualized using a heatmap. The heatmap was created using the heatmap function embedded in the ComplexHeatmap R-package [25, 26].

Differential gene expression analysis between Regression vs. PM-PC and Regression vs. Controls was performed using an un-paired limma t-test embedded in the limma R-package [27]. All comparisons were adjusted for multiple testing using the false discovery rate (FDR), and genes with FDR≤0.05 were considered as being significantly differentially expressed. The results of the differential gene expression analysis were visualized by volcano plots, using the EnhancedVolcano function embedded in the EnhancedVolcano R-package [28]. Waterfall plots were created by application of the boxplot R function and using the output from the differential gene expression analysis tests, comparing Regression with PM-PC and Regression with Controls. Specifically, the genes were sorted by the log2 fold change in descending order, and this list was visualized in a barplot, where significantly differentially up- and down-regulated genes were colored red and blue, respectively.

Gene set enrichment analysis (GSEA) was performed using the fgsea (v.1.2.8) R-package [29, 30]. GSEA was run on a list of preranked individual expressed genes using the Log2 fold change values derived from the differential gene expression analysis as ranking metric. The collection of human hallmark gene sets (https://www.gsea-msigdb.org/gsea/msigdb/human/genesets.jsp?collection=H) was used as input. The GSEA was conducted using 1,000 permutations, eps set to zero, and minimum and maximum gene set sizes were set to 15 and 500, respectively. Gene sets with FDR≤0.05 were considered as being statistically significantly enriched. A selection of significantly enriched gene sets were visualized by an enrichment plot using the plotEnrichmentData function embedded in the fgsea R-package.

### Histology and immunohistochemistry

PBs from the Regression group, PM-PC, and Controls were fixed in formalin and embedded in paraffin (FFPE). Three to four-micron sections were cut and mounted on FLEX IHC microscope slides and stained with hematoxylin & eosin (H&E). Sections were dried at room temperature and baked at 60 °C for 60 min before immunostaining. Antibodies used are shown in Supplementary Table 1. Nuclear counter staining was performed using Hematoxylin FLEX at the Dako Omnis platform. Slides were washed, dehydrated, and cover slipped using an automated Dako cover slipper (Dako/Agilent, Glostrup, Denmark).

## Results

### Patients’ characteristics

Clinical baseline data are shown in Table 1. Patients with PM-PC had a median age of 71 years (range 57–82). The Regression group had a median age of 56 (range 49–71). Median number of PIPAC treatments prior to the PB included in the Regression group was 2 (range 1–3). All patients in the Regression group had received first-line palliative systemic chemotherapy, and two patients had undergone second-line systemic chemotherapy. Patients in the Control group (chronic peritonitis due to chronic cholecystitis) had a median age of 60 years (range 19–72 years), seven females and three males, none with any known malignant disease.

**Table 1:** Baseline characteristics of patients with treatment-naïve peritoneal metastasis from pancreatic cancer (PM-PC) and PM-PC treated with systemic chemotherapy and PIPAC (Regression).

Clinical variables	PM-PC	Regression
No. of patients	10	11
Age, years, median (range)	71 (57–82)	56 (49–71)
Sex, male/female	7/3	8/3
Previous pancreatic cancer resection, n (%)	0	4 (36 %)
Number of previous PIPAC procedures, median (range)	0	2 (1–3)
Previous malignancies, n (%)	2 (20 %)^a^	3 (27 %)^b^
One-line palliative SC, n (%)	0	9 (82 %)
Two-line palliative SC, n (%)	0	2 (18 %)

^a^Myelomatosis (6 years earlier), uterine leiomyosarcoma (11 years earlier). ^b^Malignant melanoma (5 years earlier), Hodgkin’s lymphoma (36 years earlier), anaplastic oligodendroglioma (10 years earlier). PIPAC, Pressurized Intraperitoneal Aerosol Chemotherapy; SC, systemic chemotherapy.

### Unsupervised clustering analysis of mRNA expression data

**Figure 2:**
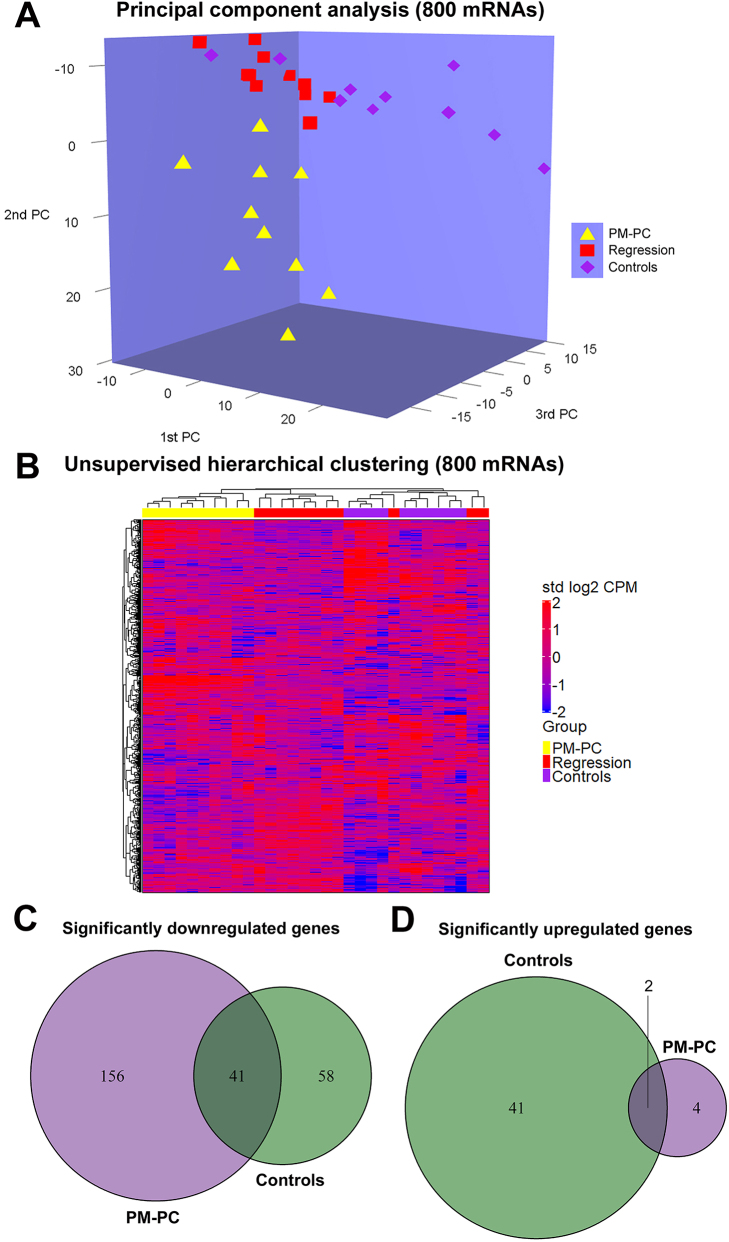
Unsupervised clustering and differential expression of RNA profiling data (800 mRNAs) from peritoneal biopsies (PBs) with chemotherapy-naïve peritoneal metastasis from pancreatic cancer (PM-PC), complete regression after systemic chemotherapy and PIPAC (Regression), and Controls. (A) Principal component analysis (PCA) plot. Clear separation between PM-PC and Controls, with accumulation of Regression cases in between. (B) Heat map based on unsupervised hierarchical clustering of all mRNAs, visualizing three clusters, where all PM-PCs accumulate in one and all Controls in another. Regression cases accumulate in a separate cluster, with a few Regression cases clustering together with Controls. (C) Venn diagram illustrating down-regulated genes in Regression (n=255). There is overlap in 41 of these genes, found when comparing Regression vs. PM-PC and Regression vs. Controls. (D) Venn diagram illustrating up-regulated genes in Regression (n=47). There is overlap in two of these genes, found when comparing Regression vs. PM-PC and Regression vs. Controls.

Based on unsupervised clustering of all mRNAs included in the mRNA profiling (n=800), we generated a principal component analysis (PCA) plot (Figure 2A). Clear separation between PM-PC and Controls was observed, with accumulation of Regression cases in between. A heat map visualized three clusters, where all PM-PCs accumulated in one and all Controls in another (Figure 2B). Regression cases accumulated in a separate cluster, with a few Regression cases clustering together with Controls. Venn diagrams, illustrating up- and down-regulated genes in Regression vs. PM-PC and in Regression vs. Controls, are shown in Figure 2C and D.

### Differentially expressed mRNAs in Regression compared to PM-PC

**Figure 3:**
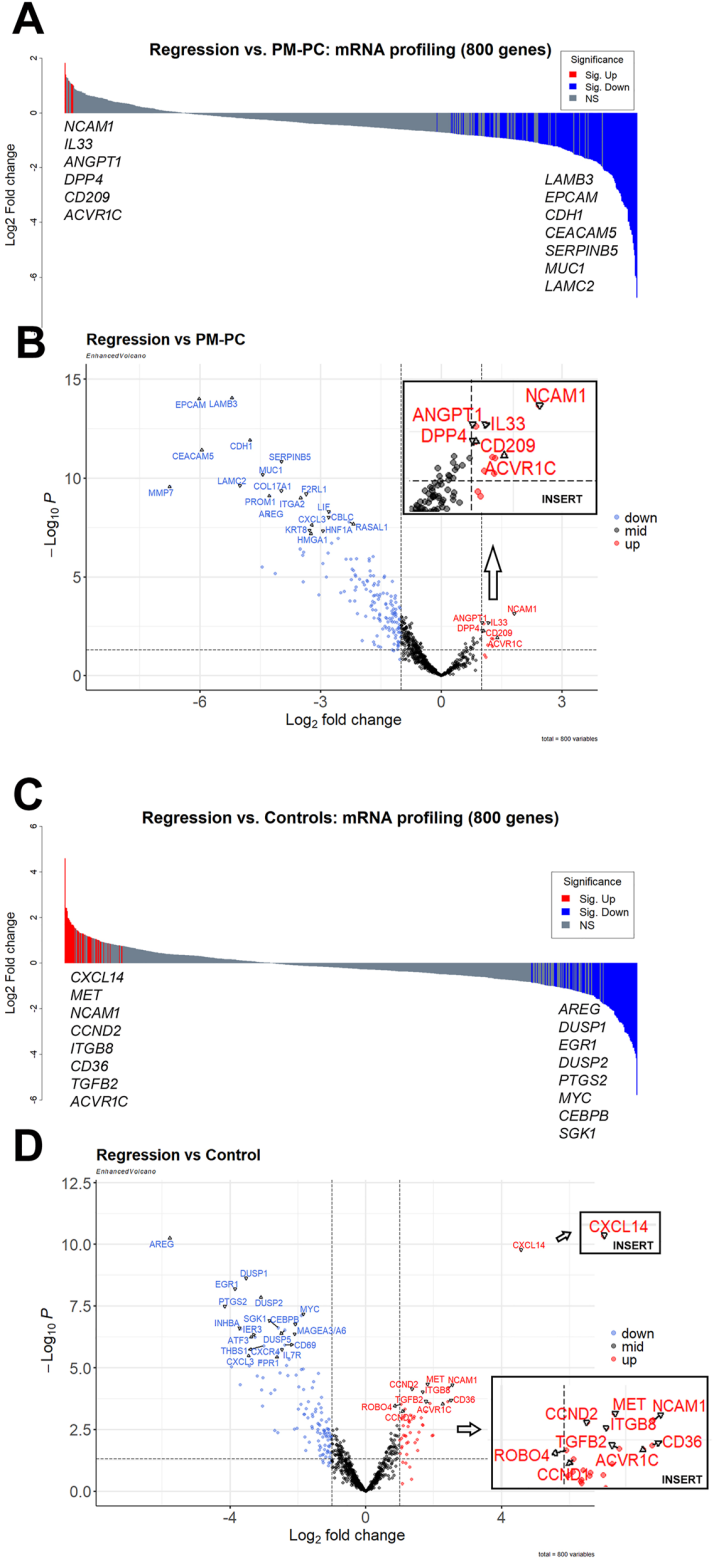
Differential expression of 800 mRNAs from peritoneal biopsies (PBs) with complete regression after systemic chemotherapy and PIPAC (Regression) compared to chemotherapy-naïve peritoneal metastasis from pancreatic cancer (PM-PC) and Controls. (A) Waterfall plot illustrating up- and down-regulated genes when comparing Regression vs. PM-PC. Significantly up- and down-regulated genes (FDR≤0.05) are shown in red and blue, respectively. Names of up to eight most significantly differentially expressed genes are given in the graph. (B) Volcano plot illustrating results of differential gene expression analysis of Regression vs. PM-PC. (C) Waterfall plot illustrating up- and down-regulated genes when comparing Regression vs. Controls. Significantly up- and down-regulated genes (FDR≤0.05) are shown in red and blue, respectively. Names of the eight most significantly differentially expressed genes are given in the graph. (D) Volcano plot illustrating results of differential gene expression analysis of Regression vs. Controls.

When comparing the mRNA expression in the Regression group with PM-PC, we found six mRNAs that were significantly up-regulated: *NCAM1*, *IL-33*, *ANGPT1*, *DPP4*, *CD209*, and *ACVR1C* (FDR≤0.05). In total, 197 mRNAs were significantly down-regulated (Table 2 and Supplementary Table 2). Figure 3A and B show a volcano plot and waterfall plot, respectively, illustrating the significantly differentially expressed mRNAs when comparing the Regression group with chemotherapy-naïve PM-PC. The significantly differently expressed genes were evaluated for over-representation of gene sets or pathways in MSigDB. When comparing with the hallmark gene sets as per gene set enrichment analysis (GSEA), TNF-α signaling via NF-kB, G2M checkpoint, epithelial-mesenchymal transition, late and early estrogen response, and coagulation were significantly down-regulated gene sets in Regression (Table 3 and Supplementary Figure 1).

**Table 2:** The six significantly up-regulated and 30 most significantly down-regulated^a^ genes when comparing Regression with treatment-naïve peritoneal metastasis from pancreatic cancer (PM-PC).

Up-regulated genes	Log2 FC	p-Value	FDR
*NCAM1*	1.81	0.0008	0.006
*IL33*	1.17	0.002	0.013
*ANGPT1*	1.05	0.002	0.014
*DPP4*	1.02	0.005	0.026
*CD209*	1.06	0.006	0.027
*ACVR1C*	1.39	0.012	0.048

Down-regulated genes^a^	Log2 FC	p-Value	FDR
*LAMB3*	−5.20	8.61E-15	3.81E-12
*EPCAM*	−6.02	9.53E-15	3.81E-12
*CDH1*	−4.75	1.20E-12	3.20E-10
*CEACAM5*	−5.95	3.75E-12	7.51E-10
*SERPINB5*	−3.97	1.49E-11	2.38E-09
*MUC1*	−4.44	7.00E-11	9.33E-09
*LAMC2*	−5.00	2.47E-10	2.74E-08
*MMP7*	−6.75	2.74E-10	2.74E-08
*COL17A1*	−3.97	4.61E-10	4.10E-08
*F2RL1*	−3.36	6.62E-10	5.29E-08
*PROM1*	−4.27	8.03E-10	5.84E-08
*ITGA2*	−3.49	1.01E-09	6.72E-08
*LIF*	−2.78	5.47E-09	3.37E-07
*AREG*	−4.29	7.57E-09	4.33E-07
*CBLC*	−2.79	9.86E-09	5.26E-07
*RASAL1*	−2.29	1.70E-08	8.51E-07
*CXCL3*	−3.22	2.45E-08	1.16E-06
*KRT8*	−3.27	4.44E-08	1.97E-06
*HNF1A*	−2.93	4.75E-08	2.00E-06
*HMGA1*	−3.24	6.58E-08	2.63E-06
*IER3*	−2.56	1.15E-07	4.38E-06
*WNT7B*	−2.72	2.05E-07	7.47E-06
*PDZK1IP1*	−3.51	3.92E-07	1.36E-05
*KRT7*	−3.43	5.47E-07	1.82E-05
*UBE2C*	−2.81	6.29E-07	2.01E-05
*CXCL8*	−3.45	8.14E-07	2.50E-05
*ZC3H12A*	−1.69	9.51E-07	2.82E-05
*ERBB2*	−1.79	1.18E-06	3.25E-05
*DTX4*	−2.11	1.21E-06	3.25E-05
*IL22RA1*	−2.37	1.25E-06	3.25E-05

^a^A comprehensive list of all significantly down-regulated mRNAs (n=197) is given in Supplementary Table 2. FDR, false discovery rate.

**Table 3:** Hallmark gene sets significantly down-regulated^a^ in Regression compared to treatment-naïve peritoneal metastasis from pancreatic cancer (PM-PC).

Down-regulated gene set^a^	Size Leading edge genes	p-Value	FDR
TNF-α signaling via NF-kB	73 *LAMB3, AREG, F2RL1, CXCL3, INHBA, LIF, IER3, CCL20, CXCL1, OLR1, PTGS2, FOSL1, CXCL2, EGR1, ZC3H12A, HES1, BIRC3, IL1A, DUSP5, EDN1, CCND1, IL7R, DUSP2, FUT4, CCL4, VEGFA, CD80, CXCL10, RELB, IRF1, ATF3, TAP1, CXCL11, CSF2, NFkBIE, MYC, IL18, IL1B, NFkB2, TNF, TNFAIP3*	1.58E-05	0.0005
Late estrogen response	22 *LAMC2, CDH1, AREG, SERPINA1, SLC7A5, CCND1, CDC20, DUSP2, FLNB*	0.0005	0.007
G2M checkpoint	18 *HMGA1, UBE2C, MKI67, SLC7A5, BIRC5, CENPF, KIF2C, EXO1, CCND1, CDC20, EZH2, BRCA2*	0.0007	0.008
Epithelial-mesenchymal transition	63 *LAMC2, AREG, MMP1, ITGA2, CXCL8, COL11A1, DKK1, INHBA, CXCL1, VCAN, COMP, IL32, FSTL3, PVR, VEGFA, SPP1, COL4A1, TNFRSF11B, THBS1, TPM1, WNT5A, CDH11*	0.001	0.007
Early estrogen response	17 *MUC1, AREG, KRT8, SLC7A5, HES1, CCND1, SLC2A1, FLNB*	0.001	0.008
Coagulation	21 *MMP7, MMP1, ITGA2, SERPINA1, OLR1, COMP*	0.003	0.014

^a^No significantly up-regulated hallmark gene sets were identified. FDR, false discovery rate. Italic values give the leading edge genes.

### Differentially expressed mRNAs in Regression compared to Controls

When comparing the mRNA expression in Regression with Controls, we found 43 mRNAs that were significantly up-regulated (FDR≤0.05) (Table 4 and Supplementary Table 3). In total, 99 mRNAs were significantly down-regulated (FDR≤0.05) (Table 4 and Supplementary Table 3). Figure 3C and D show a volcano plot and a waterfall plot, respectively, illustrating the significantly differentially expressed mRNAs when comparing Regression with Controls, which were evaluated for over-representation of gene sets or pathways in MSigDB. When comparing with the hallmark gene sets as per GSEA, interferon-α response was significantly up-regulated (Supplementary Table 4). TNF-α signaling via NF-kB, inflammatory response, epithelial-mesenchymal transition, KRAS signaling, and hypoxia were significantly down-regulated (Supplementary Table 4 and Figure 2).

**Table 4:** The 30 most significantly up- and down-regulated genes^a^ when comparing Regression with Controls.

Up-regulated genes	Log2 FC	p-Value	FDR
*CXCL14*	4.58	1.72E-10	6.86E-08
*MET*	1.82	4.67E-05	0.001
*NCAM1*	2.43	6.58E-05	0.001
*CCND2*	1.38	7.45E-05	0.002
*ITGB8*	1.68	9.63E-05	0.002
*CD36*	2.42	0.0002	0.004
*TGFB2*	1.89	0.0003	0.005
*ACVR1C*	2.28	0.0003	0.005
*ROBO4*	1.05	0.0003	0.005
*CCND1*	1.16	0.0005	0.007
*CCL14*	1.78	0.0006	0.008
*ANGPT2*	1.31	0.0008	0.010
*PECAM1*	1.15	0.0009	0.011
*TGFBR2*	1.42	0.001	0.011
*DTX4*	1.08	0.001	0.012
*WNT2B*	1.63	0.001	0.012
*WNT11*	1.41	0.001	0.012
*TNFRSF8*	1.29	0.001	0.015
*HEY1*	1.28	0.002	0.015
*IFI27*	1.29	0.002	0.016
*PPARG*	1.67	0.002	0.020
*CCL13*	1.69	0.002	0.020
*EGFR*	0.93	0.002	0.021
*SELP*	1.10	0.002	0.021
*OAS1*	1.25	0.002	0.022
*CMKLR1*	1.00	0.003	0.026
*GHR*	1.57	0.004	0.032
*CD34*	1.31	0.004	0.032
*FLNB*	0.75	0.005	0.033
*S100A4*	1.12	0.0054	0.038

Down-regulated genes	Log2 FC	p-Value	FDR
*AREG*	−5.77	5.63E-11	4.50E-08
*DUSP1*	−3.53	2.44E-09	6.50E-07
*EGR1*	−3.84	6.64E-09	1.33E-06
*DUSP2*	−3.09	1.40E-08	2.24E-06
*PTGS2*	−4.16	3.37E-08	4.50E-06
*MYC*	−1.89	7.77E-08	8.87E-06
*CEBPB*	−2.07	1.78E-07	1.78E-05
*SGK1*	−2.58	2.45E-07	2.12E-05
*INHBA*	−3.69	2.65E-07	2.12E-05
*DUSP5*	−2.38	3.03E-07	2.20E-05
*MAGEA3/A6*	−2.08	4.24E-07	2.83E-05
*IER3*	−3.26	5.82E-07	3.33E-05
*ATF3*	−3.38	5.83E-07	3.33E-05
*CD69*	−2.39	1.20E-06	6.38E-05
*THBS1*	−3.01	1.28E-06	6.38E-05
*IL7R*	−2.47	1.73E-06	8.14E-05
*CXCR4*	−2.58	2.39E-06	0.0001
*CXCL3*	−3.45	3.28E-06	0.0001
*FPR1*	−2.60	3.59E-06	0.0001
*C5AR1*	−2.25	4.53E-06	0.0002
*CXCL2*	−3.10	5.69E-06	0.0002
*VCAN*	−2.34	8.01E-06	0.0003
*COL11A1*	−3.43	8.38E-06	0.0003
*CXCL8*	−3.96	9.09E-06	0.0003
*NFIL3*	−1.85	1.11E-05	0.0003
*NLRP3*	−1.89	1.18E-05	0.0004
*CCL4*	−2.53	1.61E-05	0.0005
*FCGR2A*	−1.40	1.69E-05	0.0005
*SLC7A5*	−2.04	1.99E-05	0.0005
*TNFAIP3*	−2.29	2.07E-05	0.0005

^a^A comprehensive list of all significantly up- (n=43) and down-regulated (n=99) genes is given in Supplementary Table 3. FDR, false discovery rate.

### Immunohistochemistry

**Figure 4:**
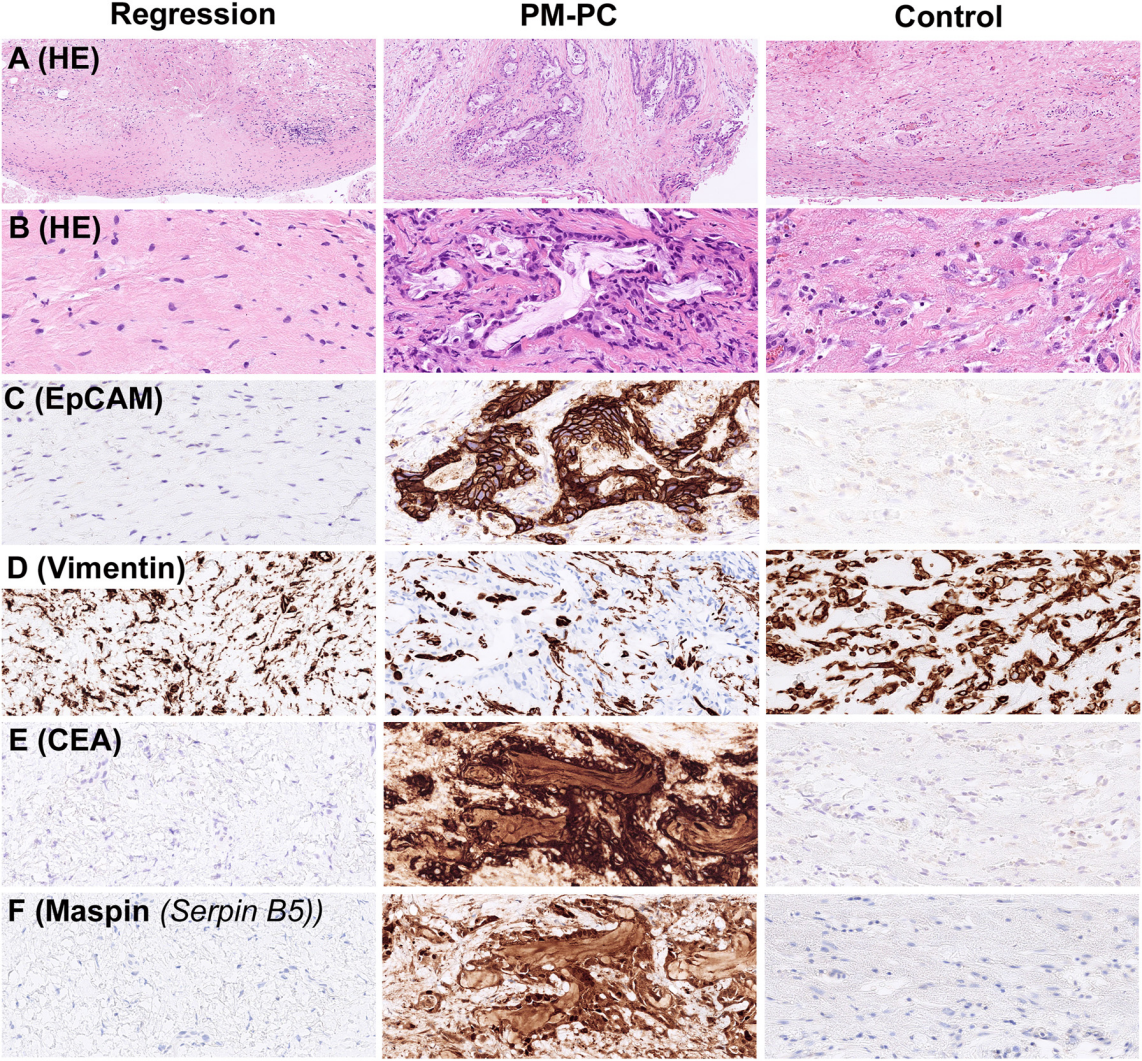
Histology and immunohistochemistry of regression after systemic chemotherapy and PIPAC (Regression), therapy-naïve peritoneal metastasis from pancreatic cancer (PM-PC), and chronic peritonitis–related fibrosis (Controls). (A) Panel showing representative specimens at low magnification (H&E, x210). (B) Panel showing representative specimens at high magnification (H&E, x710). (C) EpCAM immunostaining (x710). (D) Vimentin immunostaining (x710). (E) CEA immunostaining (x710). (F) Maspin (encoded by *Serpin B5*) immunostaining (x710).

To get an impression whether mRNA expression was related to expression of the encoded proteins, we performed immunohistochemistry for four proteins translated from four of the differentially expressed genes: CEA, EpCAM, maspin (encoded by *Serpin B5*), and vimentin. Representative images of histology of PBs with Regression, PM-PC, and Controls are shown in Figure 4A and B. EpCAM was strongly expressed in PM-PC but negative in Regression and Controls (Figure 4C). Vimentin was more strongly expressed in Regression and Controls, compared to PM-PC (Figure 4D). CEA (Figure 4E) and maspin (Figure 4F) were strongly expressed in PM-PC but negative in Regression and Controls.

## Discussion

Only few previous studies examined the transcriptomic profile of therapy-response of PM in general, and the few published studies mainly examined PM from gastric or colorectal cancer [31], [32], [33]. No such studies seem to have been published in the field of PM-PC. When comparing mRNA expression in the Regression group with PM-PC, we found six mRNAs that were significantly up-regulated (*NCAM1*, *IL-33*, *ANGPT1*, *DPP4*, *CD209*, and *ACVR1*) and 197 mRNAs that were significantly down-regulated. The down-regulated mRNAs were linked to hallmark gene sets such as TNF-α signaling via NF-kB, G2M checkpoint, epithelial-mesenchymal transition, and coagulation. When comparing the mRNA expression in Regression with Controls, we found 43 mRNAs that were significantly up-regulated, linked to the hallmark gene set of interferon-α response. We found down-regulation of 99 mRNAs, linked to TNF-α signaling via NF-kB, inflammatory response, epithelial-mesenchymal transition, KRAS signaling, and hypoxia. These differences between Regression compared to benign, unspecific peritoneal fibrosis (Controls) indicate that different mechanisms may be involved in the formation of treatment-related regressive fibrosis compared to chronic peritonitis-associated benign fibrosis.

The only previous study examining a larger number of RNAs in PM after PIPAC therapy came from Rezniczek et al. in 2016 [21]. They examined the expression of 22 genes in PM from mainly ovarian cancer taken prior to the first PIPAC (but often after several cycles of systemic chemotherapy), compared to biopsies after at least one PIPAC [21]. The post-PIPAC biopsies still contained metastatic cells, in contrast to the present study. Of the 22 examined genes, nine genes were also included in our panel (*BIRC5*, *CCNB1*, *CCNE1*, *CD44*, *MKI67*, *MMP9*, *MUC1*, *VEGF*, and *VIM*). Only *VIM* was significantly up-regulated [21]. The up-regulation of *VIM* is in agreement with our study. Of the remaining eight genes, we found down-regulation of five (*BIRC5*, *CCNB1*, *CCNE1*, *MKI67*, and *MUC1*). It seems that the down-regulation of BIRC5 and MKI67 did not reach statistical significance in Rezniczek et al.’s study. Regarding *VEGF*, we found down-regulation of *VEGF-A* and no significant dysregulation of *VEGF-B* and *-C*. Rezniczek et al. did not examine the different isoforms of *VEGF* separately. In agreement with Rezniczek, we did not find statistically significant dysregulation of *CD44* and *MMP9*.

Several studies examined the transcriptome related to PM from gastric cancer. In 2010, a 22-gene panel was identified in primary GC, predicting peritoneal relapse [34]. Wang et al. studied PM from gastric cancer after systemic chemotherapy with a multiomics approach. They did not specifically assess the transcriptomic signature in PM with histological regression but focused on differences between GC subtypes [35]. In a study from 2014, mRNA expression differences were evaluated between matched primary serous ovarian cancers and omental metastases. Pathway analysis revealed that metastatic cancer cells were more proliferatively active and less apoptotic than primary tumors [36].

Even though the histological PRGS recently was found to hold prognostic value in patients treated with systemic chemotherapy and PIPAC, we need new tools to further stratify patients and particularly to identify those patients who benefit from local therapies, such as PIPAC [2, 12, 15]. Future studies should evaluate whether RNA signatures like those identified in the present study can be used for prognostication or to predict response to therapy in patients with PM from PC or other primaries. Furthermore, it would be of clinical interest to examine whether RNA signatures can predict which patients will turn out to have long-term and short-term survival, respectively, after diagnosis of PM. The hypothesis that RNA profiling of PBs may hold clinically relevant information is also supported by the differences in RNA profile that we found not only between therapy-naïve PM-PC and Regression but also between Regression and Controls. This may indicate that fibrosis is not just fibrosis. Furthermore, future studies should evaluate whether certain markers may aid pathologists in the distinction of regressive fibrosis from structural connective tissue and unspecific fibrosis in PBs taken for assessment of therapy response.

In a previous study, we detected *KRAS* and other mutations in peritoneal biopsies and peritoneal lavage specimens from PM-PC patients before and after PIPAC treatment [5]. We found a mutation in lavage specimens in around 60 %. Graversen et al. used PCR for detection of CEA and EpCAM mRNA using peritoneal lavage fluid specimens from patients with PM of various origin including PC and reported a sensitivity of 0.88 and a specificity of 1.00 [19]. It would be of interest to examine whether large-scale RNA profiling of peritoneal fluid specimens from patients with PM is feasible and holds clinically relevant information.

Even though the PBs included in the present study were collected in the frame of two prospective studies, the PIPAC-OPC-1 and PIPAC-OPC-2 trials, the mRNA profiling performed was conducted retrospectively, which can be considered a potential limitation [2, 3]. Furthermore, we cannot know with absolute certainty whether all post-treatment biopsies in the Regression group represented the effect of systemic chemotherapy and/or PIPAC, as it cannot be entirely excluded that the fibrosis may have developed due to other causes, such as unspecific peritonitis, changes after pancreatic surgery, or antitumor immune response, in some of the PBs. However, post-treatment biopsies were taken from clips-marked areas where biopsies at baseline had shown vital tumor cells representative of PM-PC, which we consider a strength of our study. We have shown that the maximum PRGS (lowest therapy response) in clips-marked biopsies was not lower compared to a nonclips-marked biopsy from PM [20]. It is, to our knowledge, at present not possible to distinguish treatment effect that stems exclusively from systemic chemotherapy and treatment effect that stems exclusively from PIPAC. This, however, also holds true for most other anticancer treatments in oncology, when a given patient receives different treatments. Main aims were to describe the transcriptomic features of complete therapy response (PRGS-1) compared to treatment-naïve PM-PC and Controls. Therefore, we chose to exclude PBs with major (PRGS-2) and minor (PRGS-3) treatment response from the “Response” group, because such specimens still contain metastatic tumor cells.

It may be hypothesized that immunohistochemical staining for proteins encoded by genes that are dysregulated between Regression vs. Controls, for example CXL-14, may aid in the distinction of unspecific fibrosis from treatment-induced, regressive fibrosis.

In conclusion, when comparing the expression of 800 genes related to immune-oncology and stromal factors in PBs from the Regression group with treatment-naïve PM-PC, we found statistically significant up-regulation of *NCAM1*, *IL-33*, *ANGPT1*, *DPP4*, *CD209*, and *ACVR1C* and down-regulation of 197 genes linked to TNF-α signaling via NF-kB, G2M checkpoint, epithelial-mesenchymal transition, late and early estrogen response, and coagulation. We furthermore identified 142 significantly dysregulated mRNAs in Regression compared to Controls. Future studies should examine whether RNA profiling of PBs with PM from PC or other primaries holds prognostic or predictive value.

## Highlights

– Evaluation of the transcriptomic profile of treatment-naïve PM-PC compared to fibrosis due to response after systemic chemotherapy and PIPAC (Regression).
– We performed mRNA expression profiling of 800 mRNAs related to immune-oncology and tumor stroma.
– The mRNA expression profile of Regression (fibrosis after systemic chemotherapy and PIPAC) was also compared to benign chronic peritonitis-related fibrosis (Controls).
– Regression vs. treatment-naïve PM-PC showed downregulation of mRNAs related to key tumor biological pathways.
– Regressive fibrosis (Regression group) also showed considerable transcriptional differences from unspecific benign peritoneal fibrosis (Controls).

## Supplementary Material

Supplementary Material
