# Supplementary material for: RNA expression profiling of peritoneal metastasis from pancreatic cancer treated with Pressurized Intraperitoneal Aerosol Chemotherapy (PIPAC)
Source: Pleura Peritoneum. 2024 Jun 3;9(2):79–91. doi: 10.1515/pp-2024-0001 (PMC11211652; doi:10.1515/pp-2024-0001)
Supplement: Supplementary file 1 — Supplementary Material [file j_pp-2024-0001_suppl_001.pdf]

PLEURA AND PERITONEUM
SUPPLEMENTARY MATERIAL

RNA expression profiling of peritoneal metastasis from pancreatic cancer treated with systemic chemotherapy and PIPAC

Sönke Detlefsen[1,2,3], Mark Burton[3,4,5], Alan Patrick Ainsworth[2,3,6], Claus Fristrup[2,6], Martin Graversen[2,3,6,7], Per Pfeiffer[2,3,8], Line Schmidt Tarpgaard[2,3,8], Michael Bau Mortensen[2,3,6]

1: Department of Pathology, Odense University Hospital, Odense, Denmark

2: Odense Pancreas Center (OPAC) and Odense PIPAC Center (OPC), Odense University Hospital, Odense, Denmark

3: Department of Clinical Research, Faculty of Health Sciences, University of Southern Denmark, Odense, Denmark

4: Department of Clinical Genetics, Odense University Hospital, Odense, Denmark

5: Clinical Genome Center, University of Southern Denmark, Odense, Denmark

6: Department of Surgery, Upper GI and HPB Section, Odense University Hospital, Odense, Denmark

7: OPEN - Open Patient data Explorative Network, Odense University Hospital, Region of Southern Denmark

8: Department of Oncology, Odense University Hospital, Odense, Denmark

Corresponding author:
Sönke Detlefsen
Department of Pathology, Odense University Hospital
J.B. Winsløws Vej 15, 5000 Odense C, Denmark
E-mail: Sonke.Detlefsen@rsyd.dk
Phone: +45 6541 4806
Fax: +45 6591 2943

Supplementary Table 1: List of antibodies, retrieval methods, incubations times, dilutions and staining platform used for immunohistochemistry

Antigen	Species and clonality	Company	Clone	Epitope retrieval	Incu-bation	Dilu-tion	Platform	Detection
CEA	Mouse, mAb	Thermo Fisher Scientific	Col-1	HIER: CC1_32_100	20min/ 36°C	RTU	BenchMark	OptiView-DAB
EpCAM	Mouse, mAB	Nordic BioSite ApS	BS14	HIER: CC1_32_100	32min/36˚C	RTU	BenchMark	OptiView-DAB
Maspin	Mouse, mAb	Pharmingen	G167-70	HIER: CC1_32_100	32min/ 36°C	RTU	BenchMark	OptiView-DAB
Vimentin	Mouse, mAb	Ventana Medical Systems	V9	HIER: CC1_32_100	16min/ 36°C	RTU	BenchMark	OptiView-DAB

CC1: cell conditioning solution 1 (pH 8.5, Ventana Medical Systems), *CC1_X_X*: CC1_minutes incubated_degrees Celsius, *HIER*: heat induced epitope retrieval, *mAb*: monoclonal antibody, *RTU*: ready to use.

Supplementary Table 2. Significantly upretulated (n=6) and downregulated genes (n=197) when comparing Regression with therapy-naïve PM-PC

**Upregulated genes**	**logFC**	**P Value**	**FDR**
NCAM1	1.81	0.0008	0.006
IL33	1.17	0.002	0.013
ANGPT1	1.05	0.002	0.014
DPP4	1.02	0.005	0.026
CD209	1.06	0.006	0.027
ACVR1C	1.39	0.012	0.048
**Downregulated genes**	**logFC**	**P Value**	**FDR**
LAMB3	-5.20	8.61E-15	3.81E-12
EPCAM	-6.02	9.53E-15	3.81E-12
CDH1	-4.75	1.20E-12	3.20E-10
CEACAM5	-5.95	3.75E-12	7.51E-10
SERPINB5	-3.97	1.49E-11	2.38E-09
MUC1	-4.44	7.00E-11	9.33E-09
LAMC2	-5.00	2.47E-10	2.74E-08
MMP7	-6.75	2.74E-10	2.74E-08
COL17A1	-3.97	4.61E-10	4.10E-08
F2RL1	-3.36	6.62E-10	5.29E-08
PROM1	-4.27	8.03E-10	5.84E-08
ITGA2	-3.49	1.01E-09	6.72E-08
LIF	-2.78	5.47E-09	3.37E-07
AREG	-4.29	7.57E-09	4.33E-07
CBLC	-2.79	9.86E-09	5.26E-07
RASAL1	-2.29	1.70E-08	8.51E-07
CXCL3	-3.22	2.45E-08	1.16E-06
KRT8	-3.27	4.44E-08	1.97E-06
HNF1A	-2.93	4.75E-08	2.00E-06
HMGA1	-3.24	6.58E-08	2.63E-06
IER3	-2.56	1.15E-07	4.38E-06
WNT7B	-2.72	2.05E-07	7.47E-06
PDZK1IP1	-3.51	3.92E-07	1.36E-05
KRT7	-3.43	5.47E-07	1.82E-05
UBE2C	-2.81	6.29E-07	2.01E-05
CXCL8	-3.45	8.14E-07	2.50E-05
ZC3H12A	-1.69	9.51E-07	2.82E-05
ERBB2	-1.79	1.18E-06	3.25E-05
DTX4	-2.11	1.21E-06	3.25E-05

IL22RA1	-2.37	1.25E-06	3.25E-05
CEP55	-2.25	1.26E-06	3.25E-05
OASL	-1.79	1.55E-06	3.87E-05
SERPINA1	-2.95	2.09E-06	5.07E-05
CXCL5	-4.45	3.12E-06	7.35E-05
MKI67	-2.22	4.03E-06	9.22E-05
ANO1	-2.19	4.80E-06	0.0001
CCNO	-1.77	4.87E-06	0.0001
MMP1	-4.13	6.91E-06	0.0002
RRM2	-2.14	7.93E-06	0.0002
HES1	-1.68	8.67E-06	0.0002
BCL2L1	-1.76	8.85E-06	0.0002
EZH2	-1.41	8.97E-06	0.0002
FOSL1	-2.13	9.28E-06	0.0002
INHBA	-2.86	1.02E-05	0.0002
SLC2A1	-1.42	1.07E-05	0.0002
SLC7A5	-2.03	1.28E-05	0.0002
ANLN	-2.24	1.36E-05	0.0002
KIF2C	-1.85	1.50E-05	0.0003
SBNO2	-1.21	1.66E-05	0.0003
GLI1	-1.84	1.66E-05	0.0003
DUSP5	-1.55	1.81E-05	0.0003
COL11A1	-3.43	1.82E-05	0.0003
BIRC5	-1.94	1.85E-05	0.0003
EXO1	-1.66	2.19E-05	0.0003
MELK	-1.57	2.50E-05	0.0004
CDC25C	-1.92	2.51E-05	0.0004
TYMS	-1.68	2.62E-05	0.0004
CXCL2	-2.12	3.35E-05	0.0005
CENPF	-1.87	4.25E-05	0.0006
PTGS2	-2.14	4.61E-05	0.0006
WNT10A	-2.41	5.38E-05	0.0007
EGR1	-1.76	5.53E-05	0.0007
CCL20	-2.50	6.43E-05	0.0008
FANCA	-1.44	6.55E-05	0.0008
PVR	-1.35	7.18E-05	0.0009
CDC20	-1.51	7.93E-05	0.001
DKK1	-3.04	8.15E-05	0.001
BIRC3	-1.59	8.36E-05	0.001
SLC16A3	-1.65	8.41E-05	0.001
NECTIN2	-1.14	8.88E-05	0.001
CCNB1	-1.68	9.01E-05	0.001

HELLS	-1.25	9.60E-05	0.001
EDN1	-1.54	9.78E-05	0.001
SPRY4	-1.15	0.0001	0.001
FSTL3	-1.56	0.0001	0.001
FUT4	-1.25	0.0002	0.002
OLR1	-2.15	0.0002	0.002
CXCL1	-2.27	0.0002	0.002
CTLA4	-1.71	0.0002	0.002
IRF7	-1.13	0.0002	0.002
IL32	-1.66	0.0002	0.002
CCNE1	-1.26	0.0002	0.002
IFI27	-1.38	0.0002	0.002
BRIP1	-1.38	0.0003	0.002
MET	-1.73	0.0003	0.003
CDK6	-1.21	0.0003	0.003
VCAN	-1.75	0.0003	0.003
TPM1	-1.05	0.0003	0.003
IL2RA	-1.34	0.0004	0.003
CSF2	-1.02	0.0004	0.004
TPI1	-1.18	0.0004	0.004
RELB	-1.13	0.0004	0.004
TGFB3	-1.71	0.0005	0.004
ERO1A	-1.63	0.0005	0.004
FLNB	-1.23	0.0005	0.005
UBE2T	-1.15	0.0006	0.005
NECTIN1	-1.15	0.0007	0.006
H2AFX	-1.18	0.0007	0.006
IL1R2	-2.08	0.0007	0.006
BRCA2	-1.21	0.0007	0.006
IRF1	-1.13	0.0008	0.006
CCR4	-1.53	0.0008	0.006
COL4A1	-1.17	0.0008	0.007
FOXP3	-1.53	0.0009	0.007
TICAM1	-1.12	0.001	0.008
SLC11A1	-1.87	0.001	0.008
MYC	-0.99	0.001	0.008
DUSP2	-1.38	0.001	0.008
AXIN1	-0.88	0.001	0.009
FADD	-0.97	0.001	0.009
LTB	-1.67	0.001	0.009
VEGFA	-1.22	0.001	0.009
GPR160	-1.35	0.001	0.009

IL1A	-1.56	0.001	0.009
ISG15	-1.29	0.001	0.009
BBC3	-1.05	0.001	0.009
CCND1	-1.53	0.001	0.010
JAK3	-1.04	0.002	0.010
NFKB2	-0.93	0.002	0.010
CCL4	-1.25	0.002	0.011
HLA-F	-0.99	0.002	0.011
CXCR4	-1.52	0.002	0.011
TNFSF13	-0.98	0.002	0.012
IHH	-1.33	0.002	0.012
CD80	-1.21	0.002	0.012
RUNX3	-1.16	0.002	0.012
RAD51	-1.11	0.002	0.012
TNFRSF10B	-0.96	0.002	0.013
CDK2	-0.91	0.002	0.014
CHUK	-0.73	0.002	0.014
NLRC5	-0.92	0.002	0.014
WNT5A	-1.04	0.003	0.015
IFI35	-0.79	0.003	0.016
LGALS9	-1.00	0.003	0.016
PSMB10	-0.99	0.003	0.017
CTNNB1	-0.98	0.003	0.017
HRAS	-0.78	0.003	0.018
IL7R	-1.40	0.003	0.018
IRF3	-0.87	0.003	0.019
AXL	-1.04	0.004	0.020
TNFRSF4	-0.86	0.004	0.020
IL11	-1.30	0.004	0.020
TCF3	-0.87	0.004	0.020
KRAS	-1.06	0.004	0.021
TAP2	-0.98	0.004	0.021
TIGIT	-1.37	0.004	0.021
DNAJC14	-0.77	0.004	0.021
POLD1	-0.86	0.004	0.021
SNAI1	-1.00	0.004	0.021
WNT5B	-0.90	0.004	0.021
BRCA1	-0.92	0.004	0.021
PRSS1	-1.05	0.004	0.021
VTCN1	-1.22	0.004	0.022
IL2RG	-1.46	0.004	0.022
OAS3	-0.93	0.004	0.022

CASP8	-0.91	0.005	0.023
THBD	-0.94	0.005	0.023
IL2RB	-1.10	0.005	0.023
LCK	-1.29	0.005	0.025
PDCD1	-0.88	0.005	0.026
ENO1	-0.96	0.006	0.027
CD2	-1.17	0.006	0.027
TAP1	-1.03	0.006	0.029
MYD88	-0.81	0.006	0.029
SAMD9	-0.98	0.006	0.030
ALDOA	-0.88	0.006	0.030
CXCL16	-1.06	0.006	0.030
OAS1	-0.82	0.007	0.032
TGFB1	-0.82	0.007	0.032
ITGA6	-1.18	0.007	0.032
NFKBIE	-1.00	0.007	0.033
EIF4EBP1	-0.79	0.007	0.033
IRF9	-0.82	0.007	0.033
TYMP	-0.95	0.007	0.033
TNF	-0.90	0.007	0.033
STK11IP	-0.75	0.008	0.034
BAX	-0.74	0.008	0.035
PVRIG	-0.83	0.009	0.038
PSMC4	-0.87	0.009	0.038
PARP4	-0.82	0.009	0.040
RSAD2	-1.13	0.009	0.040
TAPBP	-0.93	0.010	0.041
PSMB8	-0.87	0.010	0.042
TNFRSF18	-0.92	0.010	0.042
IL1RN	-1.60	0.010	0.042
SOCS1	-0.98	0.010	0.043
CLEC5A	-1.31	0.010	0.043
E2F3	-0.76	0.011	0.044
LDHA	-0.88	0.011	0.046
PGPEP1	-0.69	0.011	0.047
ITPK1	-0.85	0.011	0.047
CD5	-1.00	0.012	0.047
TRAF1	-0.81	0.012	0.047
WDR76	-0.91	0.012	0.047
FCGR2A	-0.90	0.012	0.048
ITGB8	-1.14	0.013	0.050
HLA-B	-0.83	0.013	0.050

FDR: False discovery rate.

Supplementary Table 3. Significantly up- (n=43) and downregulated (n=99) genes when comparing Regression with Controls.

**Upregulated genes**	**logFC**	**P Value**	**FDR**
CXCL14	4.58	1.72E-10	6.86E-08
MET	1.82	4.67E-05	0.001
NCAM1	2.43	6.58E-05	0.001
CCND2	1.38	7.45E-05	0.002
ITGB8	1.68	9.63E-05	0.002
CD36	2.42	0.0002	0.004
TGFB2	1.89	0.0003	0.005
ACVR1C	2.28	0.0003	0.005
ROBO4	1.05	0.0003	0.005
CCND1	1.16	0.0005	0.006
CCL14	1.78	0.0006	0.008
ANGPT2	1.31	0.0008	0.010
PECAM1	1.15	0.0009	0.011
TGFBR2	1.42	0.001	0.011
DTX4	1.08	0.001	0.012
WNT2B	1.63	0.001	0.012
WNT11	1.41	0.001	0.012
TNFRSF8	1.29	0.001	0.015
HEY1	1.28	0.002	0.015
IFI27	1.29	0.002	0.016
PPARG	1.67	0.002	0.020
CCL13	1.69	0.002	0.020
EGFR	0.93	0.002	0.021
SELP	1.10	0.002	0.021
OAS1	1.25	0.002	0.022
CMKLR1	1.00	0.003	0.026
GHR	1.57	0.004	0.032
CD34	1.31	0.004	0.032
FLNB	0.75	0.005	0.033
S100A4	1.12	0.005	0.038
FAM124B	0.86	0.005	0.038
SFRP1	1.98	0.006	0.038
IL11RA	0.90	0.006	0.038
PLA2G2A	1.96	0.006	0.038
IFIT2	1.16	0.006	0.038
PALMD	1.18	0.006	0.038
EDN1	0.95	0.006	0.039
ITGA6	1.06	0.007	0.043
C2	0.75	0.008	0.047

CNTFR	1.51	0.008	0.048
TLR3	1.01	0.009	0.049
NT5E	0.84	0.009	0.049
MICA	0.77	0.009	0.050
**Downregulated genes**	**logFC**	**P Value**	**FDR**
AREG	-5.77	5.63E-11	4.50E-08
DUSP1	-3.53	2.44E-09	6.50E-07
EGR1	-3.84	6.64E-09	1.33E-06
DUSP2	-3.09	1.40E-08	2.24E-06
PTGS2	-4.16	3.37E-08	4.50E-06
MYC	-1.89	7.77E-08	8.87E-06
CEBPB	-2.07	1.78E-07	1.78E-05
SGK1	-2.58	2.45E-07	2.12E-05
INHBA	-3.69	2.65E-07	2.12E-05
DUSP5	-2.38	3.03E-07	2.20E-05
MAGEA3/A6	-2.08	4.24E-07	2.83E-05
IER3	-3.26	5.82E-07	3.33E-05
ATF3	-3.38	5.83E-07	3.33E-05
CD69	-2.39	1.20E-06	6.38E-05
THBS1	-3.01	1.28E-06	6.38E-05
IL7R	-2.47	1.73E-06	8.14E-05
CXCR4	-2.58	2.39E-06	0.0001
CXCL3	-3.45	3.28E-06	0.0001
FPR1	-2.60	3.59E-06	0.0001
C5AR1	-2.25	4.53E-06	0.0002
CXCL2	-3.10	5.69E-06	0.0002
VCAN	-2.34	8.01E-06	0.0003
COL11A1	-3.43	8.38E-06	0.0003
CXCL8	-3.96	9.09E-06	0.0003
NFIL3	-1.85	1.11E-05	0.0003
NLRP3	-1.89	1.18E-05	0.0004
CCL4	-2.53	1.61E-05	0.0005
FCGR2A	-1.40	1.69E-05	0.0005
SLC7A5	-2.04	1.99E-05	0.0005
TNFAIP3	-2.29	2.07E-05	0.0005
LIF	-3.20	2.20E-05	0.0006
IL1B	-3.02	2.49E-05	0.0006
ZC3H12A	-1.54	3.64E-05	0.0008
IL6	-3.69	4.90E-05	0.001
IL1R2	-1.97	5.93E-05	0.001
THBD	-1.57	6.41E-05	0.001

SNAI1	-1.83	7.70E-05	0.002
CCL3/L1	-2.68	7.81E-05	0.002
COMP	-3.06	7.90E-05	0.002
FCAR	-2.80	0.0001	0.002
JAK3	-1.30	0.0002	0.003
CTLA4	-1.69	0.0002	0.004
TPM1	-1.38	0.0003	0.005
SERPINA1	-1.91	0.0003	0.005
CCL20	-3.04	0.0004	0.005
TLR2	-1.32	0.0004	0.006
SLC16A3	-1.75	0.0005	0.007
CSF3R	-1.48	0.0005	0.007
TREM1	-1.98	0.0005	0.007
FSTL3	-1.48	0.0006	0.008
FCGR1A	-1.30	0.0007	0.009
CD40LG	-1.58	0.0008	0.010
IL10	-1.81	0.0008	0.010
CCL2	-1.80	0.0008	0.010
PNOC	-1.56	0.001	0.011
LILRA5	-1.70	0.001	0.012
TNFSF8	-1.08	0.001	0.013
KLRB1	-1.30	0.001	0.014
ITGA4	-1.16	0.002	0.016
IL24	-1.41	0.002	0.018
VEGFA	-1.39	0.002	0.020
STC1	-1.35	0.002	0.020
HSD11B1	-1.58	0.002	0.022
SLC11A1	-2.02	0.003	0.025
SELE	-1.57	0.003	0.025
ACTA2	-1.14	0.003	0.026
CCND3	-0.86	0.003	0.026
LILRA1	-1.10	0.003	0.026
CXCL5	-2.10	0.003	0.026
CD3D	-1.41	0.003	0.026
IL18R1	-1.09	0.003	0.026
ARG1	-1.24	0.003	0.027
HCK	-0.96	0.004	0.028
MMP1	-2.68	0.004	0.028
CDH11	-1.21	0.004	0.028
CCL8	-1.22	0.004	0.029
CLEC5A	-1.42	0.004	0.032
IDO1	-1.20	0.004	0.032

IL11	-2.60	0.004	0.032
GLUL	-1.08	0.005	0.033
CD2	-1.12	0.005	0.033
SAMSN1	-1.23	0.005	0.034
FOSL1	-1.34	0.005	0.034
CCR4	-1.17	0.005	0.038
NFKBIA	-1.03	0.006	0.038
CD247	-0.98	0.006	0.038
MAP3K8	-0.92	0.006	0.038
CD3E	-1.00	0.006	0.039
ICAM3	-0.86	0.006	0.039
GZMB	-1.10	0.006	0.039
ZAP70	-1.16	0.006	0.039
ITGAX	-1.26	0.006	0.039
CCL7	-1.79	0.006	0.039
CD5	-1.02	0.006	0.039
CD48	-1.03	0.008	0.047
CD45RO	-0.88	0.008	0.047
IL32	-0.99	0.008	0.048
ICOS	-0.84	0.009	0.049
ICAM1	-1.12	0.009	0.049

FDR: False discovery rate.

Supplementary Table 4. Hallmark gene sets significantly up- and downregulated in Regression compared to Controls

**Upregulated gene set**	**Size**<br>**Leading edge genes**	**p-Value**	**FDR**
Interferon-α response	42<br>*IFI27, OAS1, IFIT2, IFIT3, CXCL11, MX1, GBP4, SAMD9, IL15, CXCL10, PARP12, IFIH1, TMEM140, PARP9, EIF2AK2, HERC6, OASL*	0.001	0.006
**Downregulated gene set**	**Size**<br>**Leading edge genes**	**p-Value**	**FDR**
TNFα signaling via NF-kB	73<br>*AREG, PTGS2, EGR1, IL6, INHBA, DUSP1, CXCL3, ATF3, IER3, LIF, CXCL2, DUSP2, CCL20, IL1B, SGK1, CCL4, IL7R, CD69, DUSP5, TNFAIP3, CEBPB, MYC, NFIL3, CCL2, ZC3H12A, VEGFA, FOSL1, OLR1, TLR2, CXCL1*	1.88E-11	5.44E-10
Inflammatory response	82<br>*CXCL8, IL6, INHBA, LIF, CCL20, IL1B, FPR1, IL7R, CD69, C5AR1, MYC, NLRP3, IL10, CCL2, CCL7, SELE, CSF3R, CLEC5A, OLR1, ADM, TLR2, SELL, ICAM1, HAS2, IL18R1, CXCR6, PDPN, NFkBIA, CD48, HIF1A*	0.0004	0.005
Epithelial-mesenchymal transition	63<br>*AREG, CXCL8, IL6, INHBA, COL11A1, COMP, THBS1, MMP1, VCAN, TNFAIP3, FSTL3, VEGFA, TPM1, COL5A1, CXCL1, CDH11, ACTA2, COL3A1, CDH2, IL32, PLOD2*	0.0006	0.006
KRAS signaling up	39<br>*PTGS2, INHBA, LIF, CCL20, IL1B, CXCR4, IL7R, TNFAIP3, HSD11B1*	0.001	0.006
Hypoxia	39<br>*IL6, DUSP1, ATF3, IER3, CXCR4, TNFAIP3, NFIL3, VEGFA, COL5A1, STC1, ADM*	0.001	0.006

FDR: False discovery rate

Supplementary Figure 1.

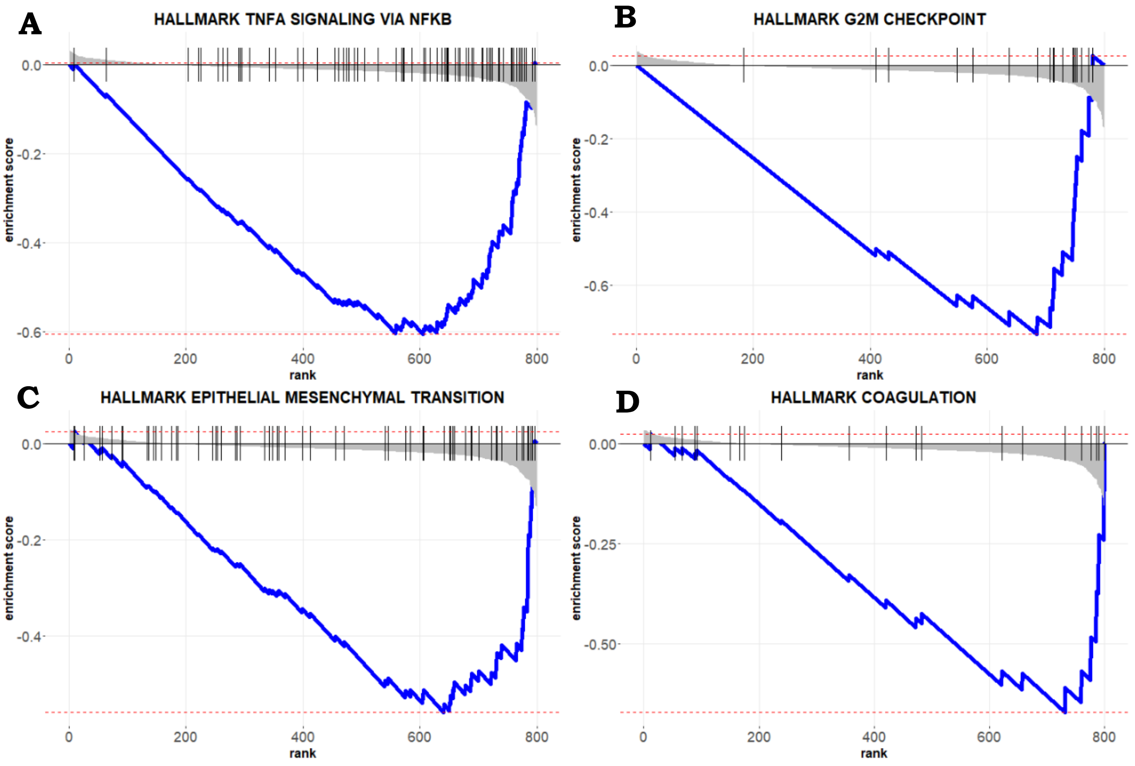

Gene set enrichment analysis curves of selected significantly differentially expressed hallmark gene sets comparing Regression vs. treatment-naïve peritoneal metastasis from pancreatic cancer (PM-PC). A. TNFα signaling via NF-kB (NES=-1.66, p=1.58E-05, FDR=0.0005). B. G2M checkpoint (NES=-1.74 p=0.0007, FDR=0.007). C. Epithelial-mesenchymal transition (NES=-1.52, p=0.001, FDR=0.008). D. Coagulation (NES=-1.63, p=0.003, FDR=0.014).

Supplementary Figure 2.

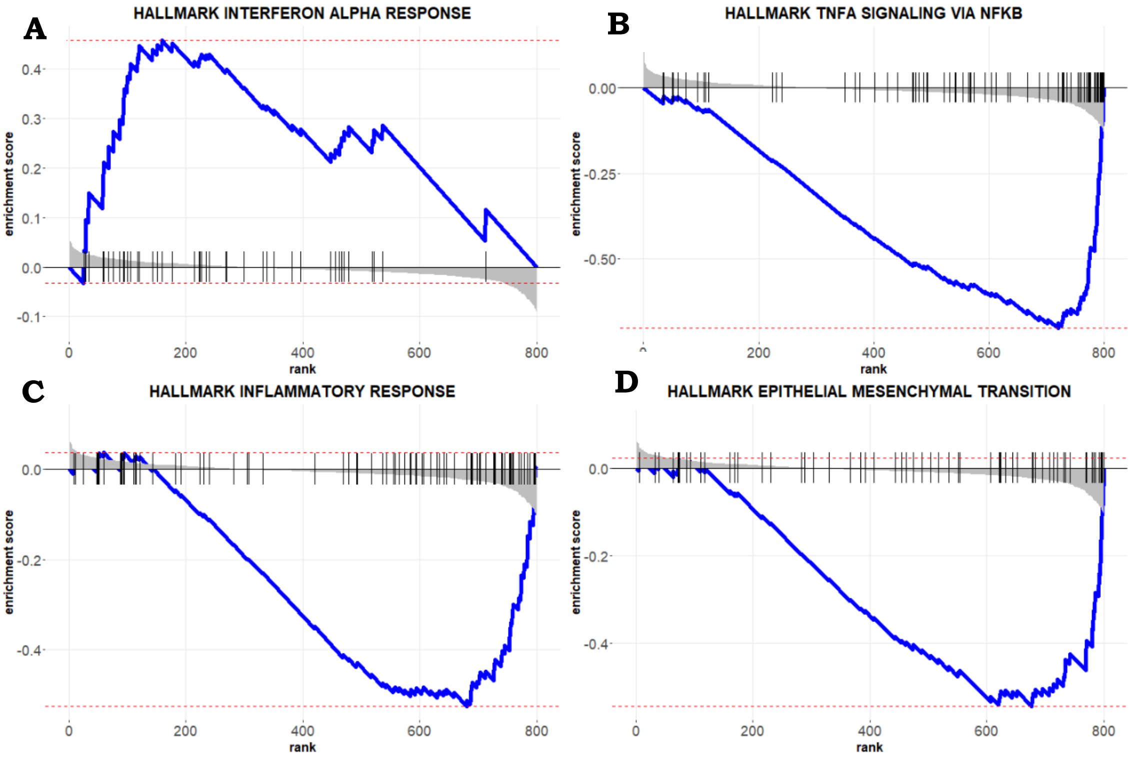

Gene set enrichment analysis curves of selected significantly differentially expressed hallmark gene sets comparing Regression vs. Controls. A) Interferon-α response (NES=1.83, p=0.001, FDR=0.006). B) TNFα signaling via NF-kB (NES=-2.22, p=1.88E-11, FDR=5.44E-10). C) Inflammatory response (NES=-1.69, p=0.0004, FDR=0.005). D) Epithelial-mesenchymal transition (NES=-1.70, p=0.0006, FDR=0.006).
